# Legionnaires’ Disease Outbreaks and Cooling Towers, New York City, New York, USA

**DOI:** 10.3201/eid2311.161584

**Published:** 2017-11

**Authors:** Robert Fitzhenry, Don Weiss, Dan Cimini, Sharon Balter, Christopher Boyd, Lisa Alleyne, Renee Stewart, Natasha McIntosh, Andrea Econome, Ying Lin, Inessa Rubinstein, Teresa Passaretti, Anna Kidney, Pascal Lapierre, Daniel Kass, Jay K. Varma

**Affiliations:** New York City Department of Health and Mental Hygiene, Queens, New York, USA (R. Fitzhenry, D. Weiss, D. Cimini, S. Balter, C. Boyd, L. Alleyne, N. McIntosh, A. Econome, Y. Lin, I. Rubinstein, D. Kass, J.K. Varma);; Wadsworth Center, New York State Department of Health, Albany, New York, USA (T. Passaretti, A. Kidney, P. Lapierre);; Centers for Disease Control and Prevention, Atlanta, Georgia, USA (J.K. Varma)

**Keywords:** Legionnaires’ disease, legionellosis, environmental exposure, disease outbreaks, epidemiology, legislation, space-time clustering, pneumonia, bacteria, bacterial infection, cooling towers, New York City, United States

## Abstract

The incidence of Legionnaires’ disease in the United States has been increasing since 2000. Outbreaks and clusters are associated with decorative, recreational, domestic, and industrial water systems, with the largest outbreaks being caused by cooling towers. Since 2006, 6 community-associated Legionnaires’ disease outbreaks have occurred in New York City, resulting in 213 cases and 18 deaths. Three outbreaks occurred in 2015, including the largest on record (138 cases). Three outbreaks were linked to cooling towers by molecular comparison of human and environmental *Legionella* isolates, and the sources for the other 3 outbreaks were undetermined. The evolution of investigation methods and lessons learned from these outbreaks prompted enactment of a new comprehensive law governing the operation and maintenance of New York City cooling towers. Ongoing surveillance and program evaluation will determine if enforcement of the new cooling tower law reduces Legionnaires’ disease incidence in New York City.

## Abstract 2

Surveillance will determine whether a new law regulating cooling towers reduces the incidence of Legionnaires’ disease.

Legionnaires’ disease (LD) is a pneumonia associated with human-made water systems and is classified as nosocomial (≈10% of cases), travel-related (≈20% of cases), or community-acquired (≈70% of cases) (*1*,*2*). LD is caused by bacteria from the genus *Legionella*, with *Legionella pneumophila* serogroup 1 (*Lp1*) being detected in up to 80% of cases (*3*). The incidence of LD has increased ≈4-fold in the United States since 2000 and ≈3-fold in Europe since 1995 (*4*,*5*). The reasons for this increase are unknown but might be partly a result of increase awareness of LD and the consequent increased testing for LD. LD outbreaks account for 4%–11% of cases; the remainder (i.e., those without a determined source of exposure) are classified as sporadic (*3*,*4*). In the United States, 62% of LD cases occur during June–October (*6*), a period of generally warm weather when commercial air conditioning systems, including those with cooling towers (CTs), are in operation. An estimated 28% of sporadic LD cases may be caused by CT emissions (*7*).

The first LD outbreak ever detected was linked to a Philadelphia hotel CT in 1976 (*8*), and since then many of the largest LD outbreaks have also been associated with CTs (*9*–*14*). Detection of LD outbreaks is made difficult by the standard medical practice of treating community-acquired pneumonia without performing diagnostic testing (*15*). When *Legionella* is suspected, the urine antigen test provides a rapid result but might miss 26% of cases (*16*). This test only detects *Lp1* and does not enable comparisons with environmental isolates (*16*). *Legionella* is a fastidious organism that requires specialized media and handling to culture. Respiratory cultures obtained after the start of antimicrobial drug use are less likely to grow; therefore, underdiagnosis and underreporting of LD cases is suspected (*6*). In addition, outbreaks of LD associated with CTs probably have gone undetected or, owing to the infrequency of obtaining clinical isolates, have been detected but not linked to a suspected CT.

In 2015, two LD outbreaks occurred in the New York City borough (county) of the Bronx, 1 of which was the largest ever in New York City and the second largest community outbreak in US history. Both outbreaks were linked to CTs by molecular characterization of clinical and environmental isolates. This prompted enactment of comprehensive legislation to regulate and inspect CTs to prevent LD outbreaks (*17*). We describe the evolution of community LD cluster detection and investigation, through the review of 6 LD outbreaks in New York City during 2006–2015, and the recent legislation enacted to control this environmental hazard.

## Methods

LD has been a reportable condition to the New York City Department of Health and Mental Hygiene (DOHMH) since 1994. Every case is investigated, by chart abstraction and patient or proxy interview using standardized questionnaires, to determine whether the exposure could be associated with a healthcare facility (nosocomial), another type of building (e.g., a correctional facility, group home, hotel, shelter, residence, or workplace), or travel. Cases not belonging to these 3 categories are considered sporadic unless a link in space or time is recognized. LD cluster detection methods using surveillance data changed during the study period; during 2006–2013, the historical limits method (*18*) alone was used, and during 2013–2015, the historical limits method was modified to reduce bias (*19*). This method compares LD cases in the last 4-week period with data from the preceding 5 years and was applied at 3 geographic resolutions (citywide, borough, and neighborhood).

In addition, analyses using building identifiers (BINs) were added in January 2013 to identify LD events of public health concern. The BIN is a unique number assigned to every building in New York City, matched to patient address, and compared with a list of healthcare and other congregate facilities (*20*). The prospective space-time permutation scan statistic (SaTScan) was added in February 2014 and is used to detect LD clustering using either the home or work address that occurs within a flexible time window (*21*,*22*).

We defined a community outbreak of LD as cases meeting the Council of State and Territorial Epidemiologists definition (*23*) that were not associated with a healthcare facility or residential building and occurred in close space and time, as defined by either a markedly elevated incidence in >1 US postal code (ZIP code) area or by >1 cluster detection systems. Assignment of patients to outbreaks was defined by residential ZIP code area, work ZIP code area, and locations visited during the incubation period, as elicited during patient interviews. To identify community outbreaks, we reviewed DOHMH LD investigation reports, related files, and surveillance data for 2006–2015. The following outbreak characteristics were summarized: borough where the outbreak occurred, ZIP code areas in the outbreak zone, onset dates of cases, number of cases, LD incidence in outbreak zone compared with the rest of New York City (using intercensal population estimate for each outbreak year by ZIP code area), number of deaths, the proportion of patients who were culture-positive for *Legionella*, environmental test results, link between environmental and clinical isolates, intervals from detection of the outbreak to environmental source decontamination, and whether an outbreak source was found.

Environmental sample *Legionella* testing was conducted by the New York State Department of Health Wadsworth Center (WC), the DOMHH Public Health Laboratory (PHL), and independent contractors. Criteria used to classify positive environmental results differed by laboratory. WC and PHL considered any culture growth as positive, whereas independent contractors used various CFU thresholds to define positive results (*24*,*25*). Culture and pulsed-field gel electrophoresis (PFGE) were performed by PHL, and real-time PCR (rPCR) and whole-genome sequencing (WGS) were performed by WC. Remediation was recommended whenever *Legionella* species known to be a risk to human health were identified. The study was determined to be public health surveillance and did not undergo institutional board review. Analyses were conducted using SAS version 9.2 (SAS Institute Inc., Cary, NC, USA) and SPSS version 22.0 (IBM Corp., Armonk, NY, USA).

## Results

During January 1, 2006–December 31, 2015, a total of 2,262 confirmed LD cases were reported in New York City residents. Six community-associated LD outbreaks, comprising 213 total cases, 207 of which were in New York City residents (9.7% of all New York City cases), occurred during the study period (Technical Appendix Figure). Three outbreaks and 84% (174/207) of outbreak-associated cases occurred in Bronx residents. Cultures were positive for *Legionella* spp. for 14.5% (30/207) of New York City resident outbreak-associated cases (all *Lp1*) and 6.3% (130/2,055) of New York City resident non–outbreak-associated cases (90 *Lp1*, 2 *L. pneumophila 3* [*Lp3*], 4 *L. pneumophila 4* [*Lp4*], 3 *L. pneumophila 5* [*Lp5*], 1 *L. pneumophila 6* [*Lp6*], 3 *L. micdadei*, and 27 *L. pneumophila* of an undetermined serogroup).

Outbreak 1 was recognized in the spring of 2006, when an epidemiologist (D.C.) noticed that several LD cases occurred in residents of a large apartment complex (>100 buildings). Twenty-nine cases occurred in the outbreak zone, consisting of 5 ZIP code areas. LD incidence in the outbreak zone during June–October 2006 was 9.7 cases/100,000 persons compared with 1.1/100,000 for the rest of New York City (Table 1). Interviews of patients failed to identify a common exposure in >35% of respondents. No patients were culture-positive for *Legionella*, and no environmental sampling was conducted at the time. Four additional LD cases occurred in the outbreak zone in a 3-week period of May–June 2007. Environmental sampling was performed on 2 supermarkets (a mister and a CT), a department store CT, and a decorative fountain. The department store CT was culture-positive for *Lp5* and a supermarket CT for *Lp3.* No source for the original outbreak or subsequent cases was determined.

**Table 1:** Community outbreaks of Legionnaires’ disease, New York City, New York, USA, 2006–2015*

NYC borough (outbreak no.)	Outbreak dates	Outbreak zone ZIP codes	No. cases			Crude rate†		Median age, y (range)	No. deaths, all cases	No. patients *Lp* culture+ (zone residents)	Environmental testing results
			Outbreak total (zone residents)	Rest of NYC		Outbreak zone	Rest of NYC				
Bronx (1)	Jun–Oct 2006	10460, 10461, 10462, 10472, 10473	29 (29)	87		9.7	1.1	57 (38–91)	1	0	No sampling performed at time of outbreak
Manhattan (2)	Aug–Sep 2008	10018, 10019, 10036	7 (7)	36		9.8	0.4	64 (46–81)	0	0	Supportive housing potable water *Lp*1, residential building potable water *L. anisa*, supermarket CT *Lp*6, *L. bozemanii*
Bronx (3)	Nov 2014–Jan 2015	10475	8 (8)	41		18.8	0.5	58 (29–69)	0	1	Residential potable water (no *Lp*1), CT 1 (27/30 culture+ for *Lp*1 and *Lp*6), CT 2 (1/10 culture+ for *Lp*6), supermarket mister (no *Lp* by PCR)
Queens (4)	Apr–Jun 2015	11354, 11355	16 (14)	26		9.2	0.3	65 (50–99)	0	0	Potable water of 2 housing complexes (6 PCR+, *Lp*2 culture+), 1 CT PCR+ and culture+ for *Lp*1
Bronx (5)	Jul–Aug 2015	10451, 10452, 10454, 10455, 10456, 10459, 10474	138 (108)	48		25.7	0.6	55 (30–90)	16	26 (23)	All (55) identified CTs in outbreak zone (14 culture+ for *Lp*1)
Bronx (6)	Sep–Oct 2015	10461, 10462, 10469	15 (10)	18		5.0	0.2	56 (31–71)	1	4	All (50) identified CTs in outbreak zone (8 culture+ for *Lp*1)


*CT, cooling tower; *Lp*, *Legionella pneumophila*; NYC, New York City; +, positive. †Cases/100,000 population.

The only community outbreak of LD detected in the borough of Manhattan occurred in the summer of 2008 (outbreak 2). An epidemiologist (D.C.) identified 7 cases clustered in space and time, and all but 1 patient was either a resident of federally subsidized (n = 2) or supportive housing for formerly homeless persons (n = 4). Two buildings were associated with 2 cases each. The LD incidence in the 3-ZIP code outbreak zone was 9.8/100,000 compared with 0.4/100,000 for the rest of New York City. Potable and hot water systems in 3 buildings, an irrigation system, a supermarket misting system, and 2 supermarket CTs were tested for *Legionella. L. pneumophila* serogroup 1 was isolated from a supportive housing building, and *L. anisa* from another residential building, both from the hot water systems. Testing of the supermarket CTs identified *Lp*6 in 1 and *L. bozemanii* in the other. No clinical *Legionella* cultures were obtained, and no definitive source of the outbreak was identified.

In the winter of 2014–2015, outbreak 3 was detected by the historical limits method, which signaled for the Bronx and was subsequently focused by a BIN analysis signal that occurred 29 days later. The cases were associated with a large apartment complex that was unique in that it had its own electricity-generating power plant that used a CT. Eight LD cases occurred over 3 months for an outbreak zone (single ZIP code area) incidence of 18.8/100,000, whereas the rest of New York City had an LD incidence of 0.5/100,000. Of note, 2 previous LD cases had occurred in this apartment complex during 2012–2013; these are not included in outbreak or rate calculation.

The outbreak marked DOHMH’s first use of rPCR to screen potential environmental sources. Four sites were sampled: the power plant CT, a mall CT, apartment building potable water, and water from a grocery store mister. Both *Lp1* and *Lp6* were identified in multiple water samples from the power plant CT by rPCR (29/30 samples) and culture (27/30 samples). The mall CT was positive for *Lp6* by rPCR (7/10 samples) and culture (1/7 samples). No *Lp1* was cultured from the potable water samples from the apartment complex; however, a consultant environmental service found *L. anisa* (2 samples from the same apartment). Samples from the supermarket mister were negative by rPCR and were not cultured. One patient isolate of *Lp1* was recovered and was shown by PFGE to be indistinguishable from an isolate from the power plant CT. The power plant CT was remediated 40 days later and identified by PFGE as the source 53 days after the outbreak was first recognized (Table 2).

**Table 2:** Timeline for investigations of community outbreaks of Legionnaires’ disease, New York City, New York, USA, 2006–2015*

Outbreak no.	Date outbreak detected	Method of outbreak detection	Outbreak source	Date implicated CT initially sampled	Date implicated tower first reported with *Lp*	Date remediation of implicated CT began	Date CT linked to human case by DNA typing	Days from *Lp* detection to start of CT remediation	Days from detection to source identification
1	2006 Oct 2	Detected by epidemiologist	ND	NA	NA	NA	NA	NA	NA
2	2008 Sep 15	Detected by epidemiologist, then by historical limits method	ND	NA	NA	NA	NA	NA	NA
3	2014 Dec 1	Historical limits method, then BIN	Power plant CT	2015 Jan 7	2015 Jan 9	2015 Jan 10	2015 Jan 23	40	53
4	2015 May 7	SaTScan	ND	NA	NA	NA	NA	NA	NA
5	2015 Jul 17	SaTScan	Hotel CT	2015 Jul 29	2015 Jul 30	2015 Jul 31	2015 Aug 12	14	26
6	2015 Sep 25	Detected by epidemiologist, then by SaTScan	Worksite CT	2015 Sep 26	2015 Sep 29	2015 Sep 29	2015 Oct 16	4	21

*BIN, building identifiers; CT, cooling tower; *Lp*, *Legionella pneumophila*; NA, not applicable; ND, not determined; SaTScan, space-time permutation scan statistic.

Outbreak 4 occurred in the spring of 2015 in a residential–commercial area in Queens. SaTScan analysis identified a cluster of 4 LD cases. The investigation included 3 LD cases in residents of 3 separate buildings of a public housing complex and 2 cases in residents of a nearby assisted-living residential building. The remaining case-patient residences were dispersed around the commercial center. Several CTs were identified in the outbreak zone, and, after visual inspection, environmental sampling was conducted at 1 CT and the residential buildings. All of the potable hot water samples from buildings in the public housing complex tested positive by rPCR and culture for *Lp2*. Potable water samples from the assisted-living facility were negative by rPCR and culture. The CT was positive for *Lp1* by rPCR and culture. Although no patient had *Legionella* infection confirmed by culture, 4 were positive from sputum samples by rPCR for *Lp1*. Because no molecular comparison of environmental and human *Legionella* isolates was possible, a definitive source of the outbreak was not identified.

The 2 Bronx LD outbreaks in 2015 included the largest outbreak in New York City (outbreak 5) and 1 in an area of the Bronx with a high density of CTs (outbreak 6). Outbreak 5 was detected by SaTScan in July and was defined by 7 ZIP codes areas (108/138 cases). Outbreak-associated cases were also found in all New York City boroughs, surrounding non–New York City counties, and in visitors from 3 other states. The incidence in the outbreak zone was highest of all the outbreaks at 25.7/100,000. The corresponding incidence in the rest of New York City was 0.6/100,000. A combined city and state effort identified 55 CTs in the outbreak zone. CTs were screened by rPCR for the presence of *Legionella*, and those that were positive for *Lp1* were cultured. Twenty-two CTs were found by rPCR to have *Lp1*, and 14 were *Lp1* culture-positive. Twenty-three (21%) of the 108 outbreak zone resident case-patients were culture-positive for *Lp1*, and the isolates were indistinguishable by PFGE and WGS to an isolate obtained from a hotel CT. No human isolate matched to another CT. The hotel CT was remediated 14 days later and identified by WGS as the outbreak source 26 days after the outbreak was recognized (Table 2).

Outbreak 6 was recognized in a section of the Bronx several miles from outbreak 5 after the latter had ended. Two cases were identified by an epidemiologist (R.S.) who recognized that a workplace of a case-patient and residence of another case-patient were on the same city block. SaTScan signaled 2 days later, identifying 10 cases in the cluster, and a total of 15 cases were included in the outbreak (Table 1). DOHMH was in the process of compiling a registry of CTs at the time and identified a high concentration in the outbreak zone (64 registered CTs). Four patient isolates grew *Lp1* and were indistinguishable by PFGE and WGS from the isolate obtained from a workplace CT. Fifty CTs were screened for *Lp1* by rPCR; 12 were positive, and 8 subsequently found to be culture-positive. The workplace CT was remediated 4 days later and identified as the outbreak source by WGS 21 days after the outbreak was recognized (Table 2).

## Discussion

The ability of DOHMH to detect and respond to community LD clusters has evolved over time. Improvements to the cadre of cluster detection tools has given DOHMH the confidence that that even small LD clusters will be detected, as in outbreak 4. Only 2 outbreaks were detected in the first 8 years of the study period, before many of the cluster detection methods were implemented or improved, whereas 4 were detected in the past 2 years, all of which signaled by 1 or more of the cluster detection systems. Cluster detection systems have shown great utility in detecting LD increases, but they are not without cost. Although we now recognize and respond to smaller LD clusters, additional investigation resources are required. After outbreak 6, case-patient work addresses were added to SaTScan, and a new daily proximity analysis, able to identify 2 or more cases occurring within 0.2 miles and 30 days of each other, was implemented in January 2016.

The ability to identify and test CTs during community outbreaks of LD also has evolved. When clusters of cases could not be linked to a building’s water system, investigators used “shoe leather epidemiology” to identify other possible sources. Decorative fountains, supermarket misters, visible CTs, and other potential sources were discovered by walking through neighborhoods and interviewing area residents. Beginning in 2015, CTs were identified based on a list of buildings that had applied for tax credits from decreased sewer use, and a New York City Department of Buildings CT list was populated from building construction permits. However, as was made clear from outbreaks 5 and 6, these lists were incomplete. The creation of a CT registry would facilitate identification of potential sources during a suspected community LD outbreak; however, the paucity of clinical cultures for environmental source comparison remains a limitation. The use of rPCR became routine in 2015 and has allowed DOHMH to rapidly screen a large number of potential sources to identify colonized CTs and request immediate remediation. For the outbreaks in which an environmental source was successfully identified, the time elapsed from the beginning of the investigation to source remediation decreased from 40 to 4 days, and the time required to identify the source decreased from 53 to 21 days. We attribute the decrease to several factors, including improved cluster detection, laboratory capacity, identification of CTs, and the experience gained from investigations involving teams of epidemiologists, laboratorians, and environmental health engineers.

DOHMH routinely communicates information about seasonal and emerging diseases to the medical community through an email listserv that includes all licensed physicians and other healthcare providers who have voluntarily subscribed. Repeated communications and media coverage during the study period regarding LD, particularly in the Bronx, likely sensitized the medical community to test patients with pneumonia for LD, as shown by the more than doubling of *Legionella* isolates obtained in outbreak-associated cases.

Although large outbreaks of LD are rare in the United States, public health officials struggle with identifying sources for the bulk of LD cases classified as sporadic. The focus has been on CTs because of their ubiquitous presence and direct discharge of potentially *Legionella* contaminated mist into the atmosphere. In New York City, 75% of the 5,886 registered CTs are located in the borough of Manhattan, but the Bronx has the second fewest (288 CTs [5%]). The concentration of CTs does not appear to predict whether or where an LD outbreak will occur. Other factors, such as CT design, maintenance, and elevation will need to be evaluated. We note, for example, rooftop CTs are, on average, at higher elevations in Manhattan than in the Bronx. The median elevation of CTs in Manhattan is 14 floors high, with 50% of the CTs located 7–46 floors high. In comparison, the median CT elevation in the Bronx is only 4 stories high, with 50% of the buildings being 2–8 stories. Higher elevation of CTs in Manhattan might present a lower risk for disease transmission, a result of greater particle dispersion, evaporation, or bacterial death; alternatively, the diffusion of contaminated mist from CTs at higher elevations might render outbreaks more difficult to detect.

Poverty probably contributes to the burden of LD because of patient susceptibility to infection, delayed access to medical care, and the maintenance of CTs, all of which play a role in LD outbreaks (*26*,*27*). The Bronx is fourth largest of the New York City boroughs by population, third by population density, and has the highest proportion of residents living in poverty (*28*–*30*). In poorer neighborhoods, the prevalences of concurrent conditions (e.g., diabetes and HIV) and smoking are elevated (*31*–*33*). In addition, building owners in poorer neighborhoods might lack the fiscal resources to hire staff or access training related to CT maintenance and implement a water safety plan for their CT.

Only 3 community-associated outbreaks, all within the last 2 years of the study period and in the Bronx, were successfully linked to a CT. In the 3 outbreaks for which no link was made, clinical isolates were not obtained. Because DOHMH does not receive negative *Legionella* culture reports, we are unable to assess how well our guidance is followed or the culture success rate. These factors remain challenges to LD source identification, control, and prevention efforts. When the first outbreak in this series occurred, no centralized registry of CTs existed. In August 2015, the New York City Council enacted a law requiring the registration, inspection, maintenance, and annual certification of CTs and other aerosol-producing engineering devices with rules promulgated by DOHMH (*17*). The rules require the creation of CT maintenance plans with routine monitoring of water quality (pH, biocide residual, and conductivity), weekly heterotrophic plate counts, weekly inspections of equipment by maintenance staff, and *Legionella* culture at least every 90 days during CT operational periods. On the basis of monitoring and sample results, specific minimum corrective actions must be made to control risk for *Legionella* amplification.

Many countries, including the United Kingdom and 9 other nations in Europe, have enacted legislation to register and regulate CTs, but no standard approach exists, and few countries perform active oversight of compliance (*34*). In the absence of oversight, compliance with regulations is often low, despite established industry standards, such as those issued by the American Society of Heating, Refrigerating, and Air-Conditioning Engineers and the Cooling Technology Institute (*35*,*36*). In New York City, unannounced inspections and financial penalties are expected to improve compliance. After the new law’s enactment, during May 9, 2016–May 31, 2017, DOHMH had inspected 3,909 (79%) of registered CT systems. Samples from 46 systems, comprising 1 or more CTs, were found with *Legionella* exceeding 1,000 CFU/mL. Remediation in all instances was performed in accordance with the new regulations. In the absence of the regulations, it is likely that no samples would have been collected and tested for *Legionella*. DOHMH would have been unaware of the potential hazards, and remediation would not have occurred.

New York City is a densely populated metropolis with infrastructure that varies from individual homes to skyscrapers. Our experience with LD investigations might not be typical of other jurisdictions, and generalizing conclusions from a series of 6 outbreaks is difficult. Because the *Legionella* urinary antigen test primarily detects *Lp1*, outbreaks caused by other strains might have occurred and were missed. However, the ubiquitous nature of *Legionella* in the environment and the rising incidence of LD nationally highlight the urgent need to shift from relying on the alarm bell of human disease to primary prevention strategies designed to limit *Legionella* colonization and dispersal from human-made aerosol-generating devices. New York City’s new CT regulations will provide a test case to evaluate whether strict CT maintenance reduces exposure to *Legionella* and reverses trends in LD incidence and outbreaks.

Technical Appendix Epidemic curves for 6 Legionnaires’ disease outbreaks, by illness onset date, New York City, New York, USA, 2006–2015.
