# Supplementary material for: Legionnaires’ Disease Outbreaks and Cooling Towers, New York City, New York, USA
Source: Emerg Infect Dis. 2017 Nov;23(11):1769–76. doi: 10.3201/eid2311.161584 (PMC5652439; doi:10.3201/eid2311.161584)
Supplement: Technical Appendix — Epidemic curves for 6 Legionnaires’ disease outbreaks, by illness onset date, New York City, New York, USA, 2006–2015. [file 16-1584-Techapp-s1.pdf]

Article DOI: https://doi.org/10.3201/eid2311.161584

Legionnaires' Disease Outbreaks and Cooling Towers, New York City, New York, USA

Technical Appendix

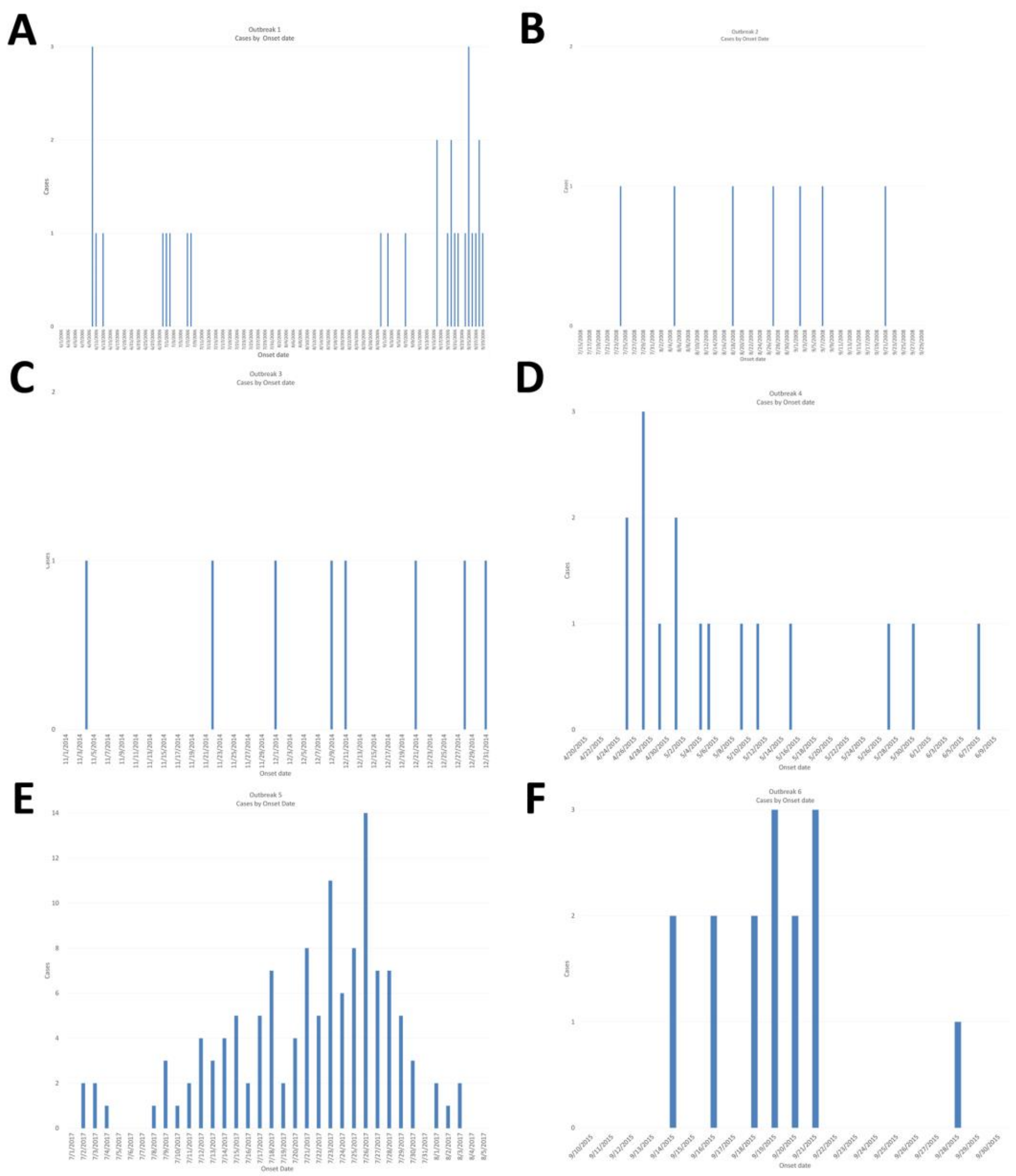

Technical Appendix Figure. Epidemic curves for 6 Legionnaires' disease outbreaks, by illness onset date, New York City, New York, USA, 2006–2015. For outbreak 5, onset dates are missing for 5 cases.
